# Retinal Mosaicking with Vascular Bifurcations Detected on Vessel Mask by a Convolutional Network

**DOI:** 10.1155/2020/7156408

**Published:** 2020-01-09

**Authors:** Xiuxia Feng, Guangwei Cai, Xiaofang Gou, Zhaoqiang Yun, Wenhui Wang, Wei Yang

**Affiliations:** ^1^Guangdong Provincial Key Laboratory of Medical Image Processing, School of Biomedical Engineering, Southern Medical University, Guangzhou 510515, China; ^2^The Information Department, The Sixth Affiliated Hospital (Gastrointestinal & Anal Hospital), Sun Yat-sen University, Guangzhou 510515, China

## Abstract

Mosaicking of retinal images is potentially useful for ophthalmologists and computer-aided diagnostic schemes. Vascular bifurcations can be used as features for matching and stitching of retinal images. A fully convolutional network model is employed to segment vascular structures in retinal images to detect vascular bifurcations. Then, bifurcations are extracted as feature points on the vascular mask by a robust and efficient approach. Transformation parameters for stitching can be estimated from the correspondence of vascular bifurcations. The proposed feature detection and mosaic method is evaluated on retinal images of 14 different eyes, 62 retinal images. The proposed method achieves a considerably higher average recall rate of matching for paired images compared with speeded-up robust features and scale-invariant feature transform. The running time of our method was also lower than other methods. Results produced by the proposed method superior to that of AutoStitch, photomerge function in Photoshop cs6 and ICE, demonstrate that accurate matching of detected vascular bifurcations could lead to high-quality mosaic of retinal images.

## 1. Introduction

Retinal images are crucial for ophthalmologists to diagnose a series of diseases. Fundus lesions caused by fundus and systemic diseases, such as diabetes, hypertension, macular lesions, fundus arteriosclerosis, and retinopathy, can appear in the retina [1]. Patients can receive timely and appropriate treatment if specialists examine and diagnose these diseases early. At present, mosaic images have been extensively used to provide a comprehensive view of the retina to assist ophthalmologists during laser surgery or other procedures. However, the retinal image captured by a fundus camera or scanning laser ophthalmoscope can only cover a local area of the eye. The retinal images captured in different field areas must be stitched together to form a mosaic image that ultimately meets the needs of the analysis of the entire area of the fundus in research and clinical diagnosis.

Numerous retinal image registration and stitching methods have been proposed in the literature. Can et al. [2–4] introduced a layered mosaicking algorithm that uses bifurcations as features of the vascular structure. However, one disadvantage of this algorithm is the occasionally indistinguishable landmarks. Ryan et al. [5] also studied the registration of retinal images using landmark correspondence. However, this approach obtains remarkably few matching point pairs. Landmark matching formulation [1] is based on retinal image alignment by enforcing sparsity in correspondence matrix. However, such approach needs a complicated computational process and high cost.

In 2010, Kwon and Ha [6] introduced panoramic video technology based on extracting important information from the image. The scale-invariant feature transform (SIFT) is one of the most robust and widely used methods. However, identifying corresponding points becomes difficult in the case of changing illumination or two surfaces with a similar intensity, as SIFT extract features using only gray information. Brown and Lowe [7] used the invariant feature for automatic mosaicking of natural images, adopting the multiresolution image fusion. Alomran and Chai [8] presented an image stitching algorithm based on speeded-up robust features (SURF), but the algorithm is restricted to an image set without exposure differences and extremely high lens distortion.

The disadvantages of methods based on SIFT or SURF include clustered detection points on the edge, uneven distribution, and insufficient effective information to describe the image. Matching of bifurcations is more appealing compared with image registration. These previous methods are limited only to images containing clearly visible vascular structures. However, the vascular structures in retinal images are often blurred when the fundus bleeds or becomes tumorous. Thus, the detection of robust features in retinal images for feature matching presents a difficulty. Simultaneously, indistinguishable features may result in matching ambiguities.

**Figure 1:**
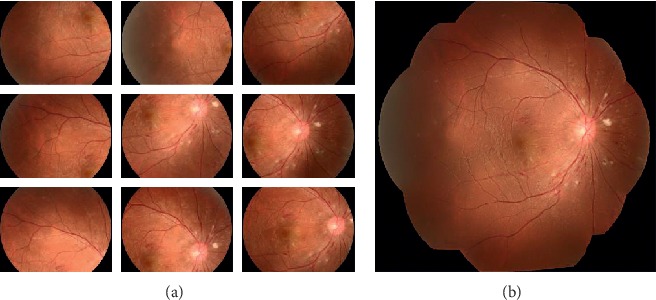
Example of a retinal mosaic image produced by our method. (a) Retinal images and (b) mosaics produced by our methods.

This work aims to improve the robustness of feature detection for indistinct vascular structures in retinal images. Numerous publications focus on using the convolutional neural network (CNN) to segment vascular structures. Jiang and Tan [9] proposed the use of conditional deep convolutional generative adversarial networks to segment the retinal vessels , by using skip connection to connect the output of the convolutional layer with the output of the deconvolution layer to avoid low-level information sharing. However, a few number of images exist in the data set to train the network, and the accuracy of vessel segmentation and the robustness of the network cannot be verified well. Alom et al. [10] proposed a Recurrent Convolutional Neural Network (RCNN) based on U-Net as well as a Recurrent Residual Convolutional Neural Network (RRCNN) based on U-Net models. 190,000 patches are randomly selected from 20 of the images as the input of networks which was a complicated computational process. Hu et al. [11] introduced a retinal vessel segmentation method based on CNN and fully connected conditional random fields. In this paper, we first employ a CNN segmentation model [12] to segment vascular structures in retinal images. Then, an effective approach is proposed to detect bifurcations as features for matching and stitching by morphology operations in the vascular structures.  Figure 1 shows an example of a mosaic image produced by the proposed method, our method for feature detection and matching benefits from previous works on retinal images.

The rest of the paper is organized as follows. First, Section 2 describes the framework and details of our proposed method.  Section 3 provides experimental results.  Section 4 discusses the results.

## 2. Method

### 2.1. Overview

**Figure 2:**
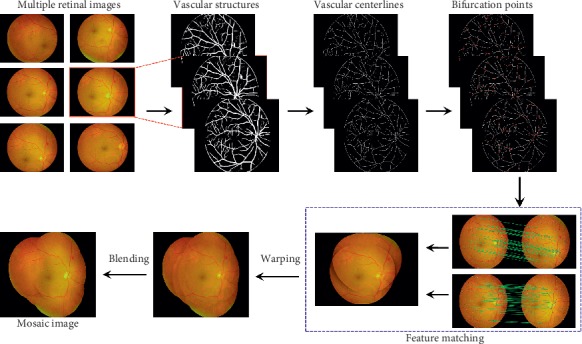
Flowchart of the proposed method for mosaic images.

This work aims to develop a practical and useful method for detecting robust and sufficient features with indistinct vascular structures and constructing an automatic mosaic of multiple retinal images. The image stitching process can be summarized by the steps shown in Figure 2. The novelty of the paper include the following:

1. The binary image of vascular structures instead of the original image was used for bifurcation detection.
2. Vascular structures of the retinal image are segmented using deep CNN model instead of traditional methods. A more accurate binary image of vascular structures can be obtained using deep CNN model.
3. Using morphology operations to locate Y- or T-form bifurcation points from the binary image which has been proved to be superior to the existing methods such as SIFT.
4. Optimal transformation parameters for stitching are obtained by further revising parameters. Then, a special coordinate mapping method was used to transform image coordinates to the same coordinate system for stitching. These steps are comprehensively described as follows. (1) Vascular structures of the retinal image are precisely segmented using a deep CNN model. The center lines of vascular structures are extracted for subsequent processing. (2) Vascular bifurcations are detected as key points by morphology operations, and their descriptors are calculated. (3) The correspondences between retinal images are established using second and nearest neighbor ratio matching [13] by calculating the Euclidean distance between feature descriptors. (4) Parameters of transformation are estimated to warp images into a uniform coordinate system using the homography matrix, which is calculated by the correspondences between image pairs. (5) The retinal images and masks are warped to uniform coordination, and vertex coordinates of images are calculated after a uniform coordinate transformation. (6) Multibland blending is used to seamlessly fuse retinal images to generate a mosaic image.

### 2.2. Feature Detection

#### 2.2.1. Segmentation of Vascular Structures

Classic methods for addressing vascular structure segmentation involve hand-crafted filters, such as line detectors and vessel enhancement techniques [12, 14–16]. Recent techniques [17, 18] are available for segmenting vascular structures of retinal images, but these techniques exhibit poor performance.

Deep CNN architectures are designed to solve the initial classification of natural images; they are also very effective for vascular structure segmentation according to the literature [12]. Maninis proposed a CNN architecture based on visual geometry group (VGG) network for retinal vascular segmentation.

A CNN model for vascular structure segmentation is already trained on the DRIVE [19] and STARE [20] datasets (40 and 20 images, respectively). The trained CNN model is publically available (All the resources of this paper, including code and pretrained models, are available at http://www.vision.ee.ethz.ch/∼cvlsegmentation/driu/). Our experiment is installed and configured Caffe on Ubuntu 14.04 with NVIDIA GPU (TitanX). Download the original DRIU model and run the model to segment the vascular structures. The precision-recall between the obtained mask and gold standard (0.822) is higher than that of the precision-recall between human-annotated result and gold standard (0.791) on the DRIVE data set. This model is employed to segment retinal vascular structures in retinal images in our experiments.

**Figure 3:**
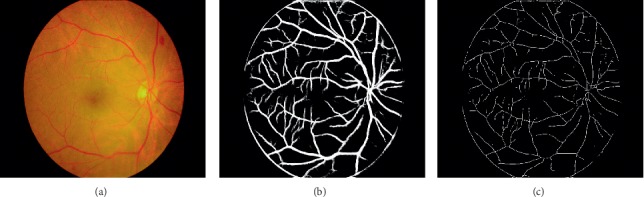
Segmentation of vascular structures and extracted vascular centerlines. (a) Retinal image, (b) Vascular structures of retina, and (c) Vascular centerlines.

Given that the size of the retinal image is inadequate for the operation of the CNN architecture, the image must be sampled before retinal vessel segmentation; the size of downsampling image is 700 × 605 pixels.  Figure 3(a) shows a retinal image, and Figure 3(b) is the segmented retinal vascular structures obtained using the CNN model. We will further research how to improve the model to obtain the resulting output without boundary.

Subsequently, a fast parallel algorithm [21] is applied for thinning images to extract the center lines of retinal vessels segmented by the CNN model. This algorithm extracts the center lines of an image through the removal of image contour points, except for points belonging to the skeleton. Iteration over several times will obtain the final skeletons as the vascular centerlines (e.g., Figure 3(c)).

#### 2.2.2. Bifurcation Detection and Descriptors

**Figure 4:**
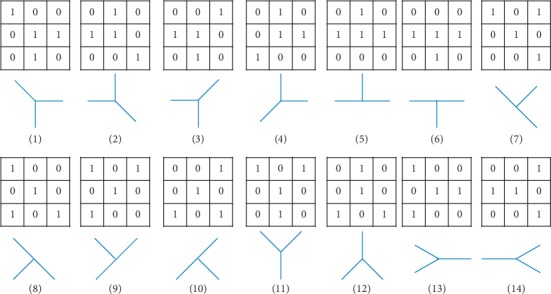
3 × 3 Y- and T-form structural elements.

Bifurcations of vascular centerlines are detected as matching features. Analysis of geometrical structures of bifurcations is important to design a scheme to solve this problem. From the observations, the vascular branch and crossover are generally presented as Y- or T-form, respectively. Hence, we use morphological operations to locate bifurcation points with a set of structural elements. Multiple Y- or T-form structural elements are applied to erode the vascular centerlines mask. The structure element of size 3 × 3 is selected according to the literature [22]. The last four Y-form structural elements shown in Figure 4 include most vascular bifurcations by comparing the various structural elements to erode the same binary image in our experiments. Finally, 14 structure elements are selected from a set of structural elements to erode vascular centerlines images to produce the highest number of correctly detected bifurcations and no falsely detected features. More criteria will be considered for selecting structural elements in future work.

One of the structural elements (Figure 4) is used to erode the vascular centerlines image, which will result in an eroded image containing numerous discrete points.

A total of 14 images are produced during image erosion by 14 structural elements. An existing problem is the addition of the 14 images. We add up all eroded images in a certain weight. The function is defined as follows:

(1) $$\begin{array}{c} C_{1}=\frac{1}{2}\left(A\Theta B_{1}\right)+\frac{1}{2}\left(A\Theta B_{2}\right)\\ \mathrm{for}\;i=2\;\mathrm{to}\;13\;\mathrm{do}\\ \;C_{i}=\frac{i}{i+1}C_{i-1}+\frac{1}{i+1}\left(A\Theta B_{i+1}\right)\\ \mathrm{end}\;\mathrm{for} \end{array}$$

where *B*_*i*_ denotes the 3 × 3 structural element, *B*_1_, *B*_2_,…, *B*_14_ contains all 14 structural elements. For example, *C*_1_ represents the first image that adds the image eroded by *B*_1_ and that by *B*_2_ together with 1/2 weight. Similarly, *C*_2_ represents the image that adds image *C*_1_ and the image eroded by *B*_3_. *C*_13_ is the ultimate image that adds the 14 eroded images with weight 1/14.

**Figure 5:**
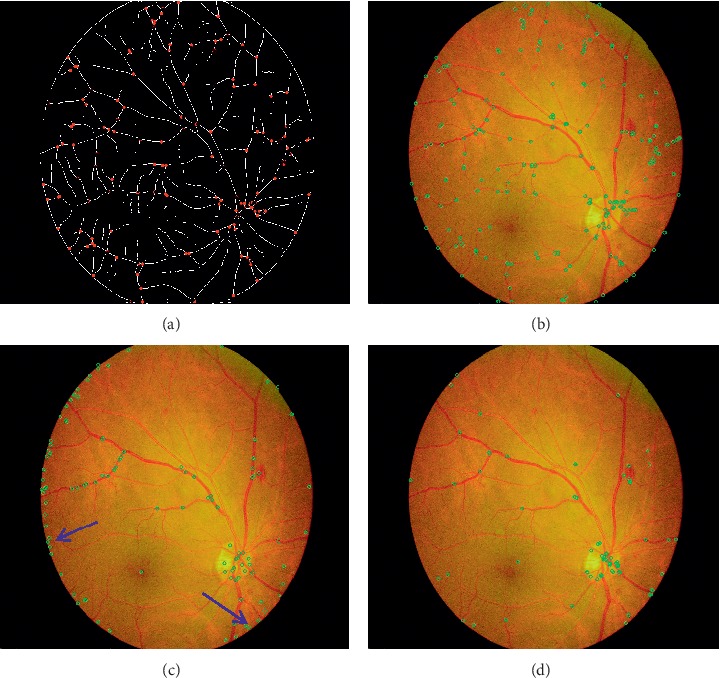
(a) Features (in red) detected by our algorithm. (b) Features marked in green dots on the retinal image. (c) and (d) Several features detected by SIFT and ORB, respectively, only concentrating on the edge.

A binary image is obtained by adding all eroded images, in which the white points with the gray value of 255 are the initially detected features. The vessel bifurcation presented as cross-form may be satisfied with multiple Y-form structures. Therefore, several subpixel points around the vessel bifurcation may be detected as features by different structural elements. Only one of these points is taken as the vessel bifurcation point in the ultimate binary image. Iterating through each pixel in binary image, if a pixel point with a gray value of 255, it sets the grayscale value of the 5 × 5 neighborhood on the point to 0. Finally, the features are expected to demonstrate vascular bifurcations. The coordinates of these features are marked in the vascular centerlines image, as shown in Figure 5(a). The operation on the gray image only locates the position of the feature. Then, feature matching is conducted on the original retinal image.  Figure 5(b) shows the marked features in the original retinal image.

Compared with features detected in the original retinal image using SIFT algorithm (Figure 5(c)), the SIFT features are mainly concentrated on the edge and are low in number. Similarly, several features are detected in the original retinal image using oriented FAST and rotated BRIEF (ORB) (Figure 5(d)). We apply different feature detection algorithms, including SIFT, ORB, and SURF, on 62 fundus images and calculate the average running time and the number of detected features. As listed in Table 1, SIFT algorithm spends the longest time, and SURF contains a number of features. Although SURF acquires the maximum number of features, this algorithm is time consuming and mainly focuses on one area, which is easily noticed in feature matching in the next section. Trading off between the running time and number of features, our feature detection algorithm is superior to other feature detection algorithms.

Numerous bifurcations will be produced after feature detection. A descriptor is then constructed to describe the distribution of intensity content within the feature neighborhood. Similar to the gradient information extracted by SIFT [23] and its variants, SURF [24] builds on the distribution of first-order Haar wavelet responses in *x* and *y* directions rather than the gradient and uses only 64 dimensions. SURF first calculated the Haar wavelet responses in *x* and *y* directions within a circular neighbourhood of radius 6*s* around the interest point, with *s* the scale at which the interest point was detected. The dominant orientation is estimated by calculating the sum of all responses within a sliding orientation window of size π/3. The horizontal and vertical responses within the window are summed. The two summed responses then yield a local orientation vector. The longest such vector over all windows defines the orientation of the interest point.

A square window centered around a feature was constructed and oriented along the orientation we already got above. The size of the window is set to 20*s*. The region is split up regularly into small 4 × 4 square subregions. For each subregion, Haar wavelet responses are computed at 5 × 5 regularly spaced sample points. The Haar wavelet responses in horizontal and vertical directions (filter size 2 s) are represented as *d*_*x*_ and *d*_*y*_, respectively. Then, wavelet responses *d*_*x*_ and *d*_*y*_ are summed up over each subregion as ∑*d*_*x*_ and ∑*d*_*y*_, respectively. The sum of the absolute values of responses (|*d*_*x*_| and |*d*_*y*_|) is also extracted to obtain information regarding the polarity of intensity changes. Hence, each subregion has a 4D descriptor vector *v* for its underlying intensity structure *v* = (∑*d*_*x*_, ∑*d*_*y*_, Σ|*d*_*x*_|, Σ|*d*_*y*_|). Concatenation for all 4 × 4 subregions results in a descriptor vector of length 64. Once the feature descriptors have been calculated, the correspondences between all image pairs can be established by calculating the Euclidean distance of those descriptors.

### 2.3. Transformation Estimation

#### 2.3.1. Bifurcation Matching

The matched candidate of each feature is determined by identifying its nearest neighbor in the features from the target image [23]. The nearest neighbor refers to the feature with minimum Euclidean distance of the descriptors. We obtain the descriptor of a bifurcation in image *I*_*i*_ and calculate its Euclidean distances to the descriptors of all features in another image *I*_*j*_. An effective measure is obtained by comparing the distance of the closest neighbor to that of the second-closest neighbor to determine correct matching. To suppress matches that could be regarded as possibly ambiguous, matches with a distance ratio larger than 0.3 will be rejected [25]. One of the bifurcation matching parameters was customized. This parameter is the confidence that indicates two images come from the same mosaics. The default value is 1.0. In our user interface, the confidence was set to an option to choose a number between 0.5 and 1.5 freely to generate different mosaics.

Each pair of potentially matching images includes a set of feature matches that are geometrically consistent (inliers) and another set of inconsistent features (outliers) inside the area of overlap. Random sample consensus (RANSAC) [7, 26] can be used to select a set of inliers that are compatible with a homography between images. Let *X* be a point on the retina with projections *x*_*i*_ and *x*_*j*_ in two images taken from different angles. A homography matrix **H** describes the transformation that connects *x*_*i*_ and *x*_*j*_ for any point *X* on the retina, *x*_*j*_ = **H ***x*_*i*_. The inliers or outliers are classified by the distance threshold *t*. When ‖*x*_*j*_ − **H***x*_*i*_‖ > *t*, the matching point is considered as outliers, *t* = 3 and the number of iterations is 2000. Then, it can get a pure sample with probability *p*=0.99. The optimal homology matrix **H** is obtained.

**Figure 6:**
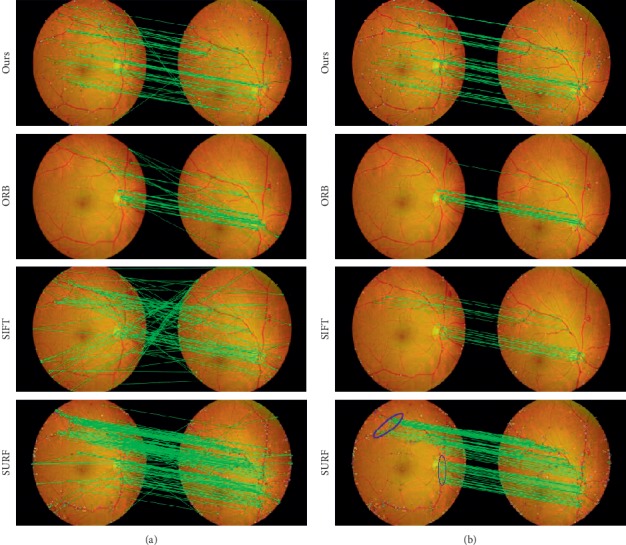
Matching result images of evaluation and comparison for different algorithms. The first column shows the initial matching result, whereas the second column depicts the result obtained after the elimination of outliers using RANSAC. (a) Initial matching result and (b) matching result after eliminating outliers by RANSAC.

Figure 6 shows the correspondence between different features of an image pair. The first, second, third, and fourth rows indicate our algorithm, ORB, SIFT, and SURF, respectively. The number of matching points of ORB and SIFT is less than that in our algorithm. Otherwise, although SURF contains more matching points, it mainly concentrates on certain areas drawn by a blue circle. These points contain little information as they occupy a small number of retinal vessels.

#### 2.3.2. Image Warping

The next step estimates transformation through the matching of detected bifurcations for retinal image pairs. Assuming that the camera rotates around the center of the optical axis, the transformation model of the image is estimated. This section first introduces how to use the homography matrix to calculate the parameters of the transformation model and then uses these parameters to transform image coordinates to the same coordinate system and perform image stitching.

Assuming that the camera rotates around its central axis, the transformation model of the image is defined as follows:

(2) $$\begin{array}{c} T_{2}\left(p;R,K\right)=RK^{-1}p. \end{array}$$

Each image has a camera intrinsic parameter matrix *K* and an extrinsic matrix *R*. *K* is a 3 × 3 intrinsic parameter matrix, $K=\left[\begin{array}{ccc} f_{x} & 0 & c_{x}\\ 0 & f_{y} & c_{y}\\ 0 & 0 & 1 \end{array}\right]$. *f* is the focal length of the camera and (*c*_*x*_, *c*_*y*_) is the center point coordinates of the image. Estimation of focal length *f* [27]is as follows:

(3) $$\begin{array}{c} f_{0}^{2}=\frac{h_{23}^{2}-h_{13}^{2}}{h_{11}^{2}+h_{12}^{2}-h_{21}^{2}-h_{22}^{2}},\;\text{if }h_{11}^{2}+h_{12}^{2}\neq h_{21}^{2}+h_{22}^{2}\\ \text{or }f_{0}^{2}=-\frac{h_{13}h_{23}}{h_{11}h_{21}+h_{12}h_{22}},\;\text{if }h_{11}h_{21}\neq -h_{12}h_{22}, \end{array}$$

where *h*_11_, *h*_12_,… are elements of the homography matrix **H**. Calculate *f* of two images from images set and take the median of *f* of all image as the focal length of all images.

*R* is a 3 × 3 extrinsic matrix. The matrix *R* of the reference image is the identity matrix. *R* can be roughly estimated according to the intrinsic parameter matrix and the homography matrix, *R*_*j*_=*R*_*i*_*K*_*i*_^−1^*H*_*ij*_^−1^*K*_*j*_. Given the matrix *R* of an image, intrinsic parameter matrix *K*_*i*_*K*_*j*_ of the two images, and the homography matrix **H** of the two images, the matrix *R* of another image can be obtained. All image camera parameters are revised by beam adjustment. The matching points of pairs of images are mapped into the three-dimensional space, calculating the camera parameters with the minimum sum of squares of matching errors. The function is defined as follows:

(4) $$\begin{array}{c} \left(R,K\right)=\min{\underset{p\in P,q\in Q}{\sum }\left\|R_{i}K_{i}^{-1}p-R_{j}K_{j}^{-1}q\right\|}^{2}, \end{array}$$

where *R*_*i*_*K*_*i*_^−1^=*R*_*j*_*K*_*j*_^−1^*H*_*ij*_. Let the matching points of pairs of images be denoted as *P* and *Q*. An initial correspondence set (*p*_*i*_, *q*_*i*_) ∈ *C*_0_ containing all matching points is established. Levenberg-Marquardt [28] algorithm is used to solve the function, and the optimal parameters *R* and *K* are obtained.

The parameters of the transformation model are used to transform the coordinates of all stitched images to the same coordinate system. Coordinate transformation is performed on the image in the following steps:

1. Suppose the point (*x*, *y*, *z* = 1) of the initial image *T* is projected to the same coordinate (*x*′, *y*′, *z*′). According to *K*, *R*, there is a relation as follows:

(5) $$\begin{array}{c} \left[\begin{array}{c} x^{\prime }\\ y^{\prime }\\ z^{\prime } \end{array}\right]=RK^{-1}\left[\begin{array}{c} x\\ y\\ z \end{array}\right]. \end{array}$$

(2) Mapping the coordinate in (1) to the spherical coordinate system (*u*, *w*, *v*). The relationship is as follows: *u* = atan (*x*′/*z*′), $w=y^{\prime }/\sqrt{x^{\prime 2}+y^{\prime 2}+z^{\prime 2}}$, *v*=*π* − acos *w*.
(3) The coordinate is backprojected onto the warped image by means of backprojection. First, the coordinates (*x*′, *y*′, *z*′) are obtained by the inverse transform for spherical coordinates in (2): *x*′=sin *v* × sin *u*, *y*′=−cos *v*, *z*′=sin *v* ×  cos *u*. Then the coordinates of the warped image are obtained by backprojection:

(6) $$\begin{array}{c} \left[\begin{array}{c} x\\ y\\ z \end{array}\right]=KR^{-1}\left[\begin{array}{c} x^{\prime }\\ y^{\prime }\\ z^{\prime } \end{array}\right]. \end{array}$$

Finally, the coordinates of the initial image *T* are transformed to the same coordinate system.

#### 2.3.3. Image Blending

When all images are warped into the same coordinate system, the images are blended to construct a mosaic image with invisible image boundaries [29]. Each sample (pixel) at the same location should exhibit the same intensity in the images for mosaics. However, no such case is observed in actual situations. After gain compensation, several image edges remain visible due to several causes, such as vignetting (decreasing intensity toward the edge of the image), misregistration of the mosaicking procedure, and radial distortion [7]. In 2D, the work of Burt [30] and Adelson on multiband blending has proven particularly effective for image mosaicking without blurring and ghosting artifacts. Suppose image *A* and image *B* are images to be blended. The method includes the following steps:

1. Gaussian pyramid is constructed according to images *A* and *B,* respectively: *G*_*A*0_, *G*_*A*1_, *G*_*A*2_, *G*_*A*3_, *G*_*A*4_ and *G*_*B*0_, *G*_*B*1_, *G*_*B*2_, *G*_*B*3_, *G*_*B*4_. The construction of Gaussian pyramid is divided into two steps: Gaussian smoothing and downsampling. The layers of Gaussian pyramid are 5. Gaussian kernel of size 5 × 5. The frequency band of the two adjacent layers decreases by 1/8 times and the width and height of the image reduces by 1/2 times.
2. Laplace pyramid is constructed, respectively, by subtracting adjacent two layer Gaussian pyramid: *L*_*A*0_, *L*_*A*1_, *L*_*A*2_, *L*_*A*3_, *L*_*A*4_ and *L*_*B*0_, *L*_*B*1_, *L*_*B*2_, *L*_*B*3_, *L*_*B*4_
3. The Laplace pyramid at the same level is blended by mask, denoted as *L*_*C*0_, *L*_*C*1_, *L*_*C*2_*L*_*C*3_, and *L*_*C*4_
4. The Laplacian pyramid of the upper layer is sampled to the same resolution as *L*_*C*0_
5. The images obtained in 4 are superimposed to obtain a final output image *C*.

**Figure 7:**
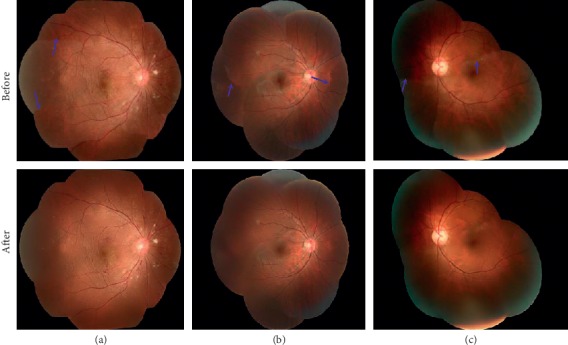
The mosaicking before and after the application of the image blending method. The first row shows the mosaics before the image blending, whereas the second row depicts the result after the image blending.

Figure 7 shows the comparison of four sets of mosaic images before and after image blending. The first row shows mosaic images with visible image boundaries (blue arrows). The second row shows mosaic images after the application of the multiband blending. The boundary of the overlapping areas of the image transitions naturally, and the quality of the mosaic image has been greatly improved.

### 2.4. Evaluation Metrics

The root mean square error (RMSE) [31] has been used as a standard statistical metric to measure the matching performance of our algorithm. Suppose *n* samples are used to calculate matching errors (*e*_*i*_, *i* = 1, 2,…, *n*). RMSE is calculated for the data set as shown in the following:

(7) $$\begin{array}{c} \text{RMSE}=\sqrt{\frac{1}{n}\sum_{k=1}^{n}e_{k}^{2}}. \end{array}$$

Following a certain order, the errors *e*_*k*_, *e*_*k*_=*R*_*i*_*K*_*i*_^−1^*p*_*k*_ − *R*_*j*_*K*_*j*_^−1^*q*_*k*_, *R*_*i*_, *K*_*i*_, *R*_*j*_, *K*_*j*_, which can be known from equation (4), *k* = 1, 2,…, and *n* can be written into an *n*-dimensional vector, where *n* denotes the total number of matching points from two images. For each matching point (*p*_*k*_, *q*_*k*_) ∈ *C*_0_, according to equation (7), the RMSE of the matching points from two images is calculated. A low value of RMSE indicates desirable matching.

The feature matching evaluation also refers to the concept of recall rate [13, 32], which is a well-known performance metric. Using the recall rate as the metric to evaluate the matching performance is mainly from the inspiration of this reference [32]. The recall rate is defined as follows:

(8) $$\begin{array}{c} \text{Recall}=\frac{\text{retrieved}\_\text{correct}\_\text{matches}}{\text{all}\_\text{correct}\_\text{matches}}, \end{array}$$

where retrieved_correct_matches denote the number of correct matching points obtained using our matching algorithm. All_correct_matches are manually marked by experts in the reference and target images.

## 3. Experiments

### 3.1. Image Data

We implemented the proposed method in C++ with OpenCV library. The experiments were performed on a PC with 8 GB RAM and an Intel i5-6500 CPU (3.20 GHz). The data set, which includes 62 retinal images, was collected from the hospital in Guangdong, China. Images are obtained from 14 different eyes. A total of 2–9 images are acquired for each eye. The image format is JPG format. The original size of images in the data set is inconsistent, including 2304 × 1728 and 1600 × 1200. A total of 190 image pairs with overlaps are used to evaluate the performance of feature matching.  Table 2 provides a detailed description of the overlaps between image pairs. In the experiment, several stitching software were compared with the proposed method. The stitching software included AutoStitch, photomerge function in Photoshop cs6 and ICE (Image Composite Editor).

### 3.2. Comparison of Feature Matching Methods

The experiment is conducted to investigate the effect of the overlap ratio of image pairs on the matching error.  Table 2 lists 10 pairs of retinal images from the data set and shows matching errors with different overlap ratios. In general, desirable matching can generally be achieved when the overlap ratio of image pairs is high. The number and spatial distribution of matched features are important factors that affect registration results. From experiment results, if an image pair exhibits a high overlap ratio but contains several detected vascular bifurcations on the images or unevenly distributed detected bifurcations, then a high matching error would be obtained. The average root mean square error of all image pairs is 1.82 pixels. To measure the performance of the matching algorithm more accurately, we plan to compare the registered image coordinates with the ground truth coordinates on more manually annotated samples.

Table 3 lists the quantitative matching performances of different features. The numbers of matched points for ORB, SIFT, SURF, and our method total 29, 17, 42, and 62, respectively. Although the number of matched points obtained by SURF is close to that of our matching method, SURF spends much more time (0.689 s) than our proposed method (0.249 s). ORB and SIFT consume less time but have fewer matched points than our algorithms. Our method found more correct matches than approaches such as SIFT or SURF. Vascular bifurcations obtained by our method are more evenly distributed than features obtained by SIFT as shown in Figure 5. In the meanwhile, matching points obtained by our method are more evenly distributed than SIFT or SURF according to the matching results. In this case, having more matches leads to better mosaic.

**Figure 8:**
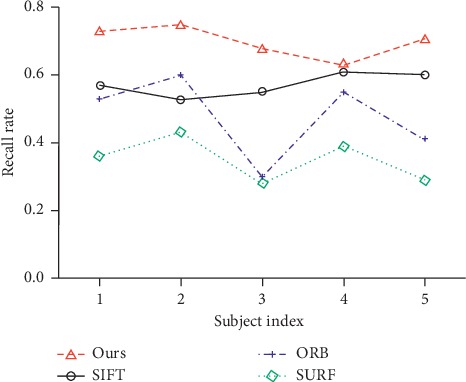
Recall rate of paired retinal images for different matching algorithms.

Figure 8 compares the recall rate of different features for image pairs. This figure lists five pairs of retinal images from the data sets and shows the quantitative results of the recall rate. As shown in Figure 8, an optimal recall rate is obtained using our registration method. Although the number of matching points obtained by SURF is close to that of our matching method, the average recall rate obtained by our matching method is considerably higher than that of SURF (0.70 to 0.35). The matching recall rate produced by ORB is lower than that of SIFT (0.47 to 0.57). Therefore, the method proposed in this paper performs better than other algorithms according to the statistics of the aforementioned indicators.

### 3.3. Qualitative Evaluation

**Figure 9:**
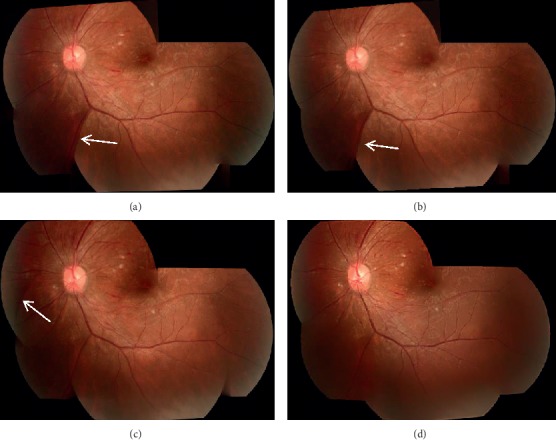
Results of four different stitching methods: (a) ICE; (b) Photoshop cs6; (c) AutoStitch; (d) proposed method.

Figure 9 shows the mosaic image stitched by four methods. a∼d are the results of ICE, photomerge function in Photoshop cs6, AutoStitch, and our method, respectively. Figures 9(a) and 9(b) both have mismatched vascular structures, as shown in the white arrow. The visible boundary between the stitched image in Figure 9(c) and the image transition is unnatural. In contrast, the mosaic image produced by our method possesses the highest similarity with the original image.

**Figure 10:**
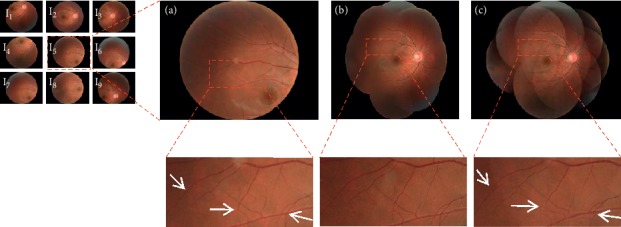
Comparison of mosaic images. Left: retinal images captured from one retina. (a) Magnified source image from sample data. (b) Mosaics produced by the proposed method. (c) Stitching result obtained by AutoStitch. Regions in red rectangles are zoomed in to display details. Three vascular mismatching structures are indicated by white arrows in (c).

**Figure 11:**
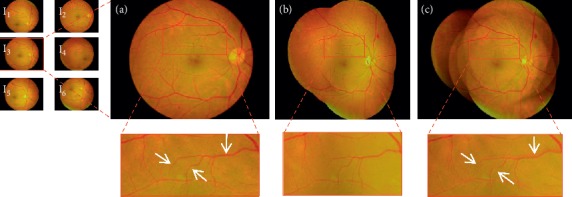
Detailed comparison of mosaic images produced by different methods. (a) Original retinal image. (b) Mosaic image produced by the proposed method. (c) Stitch result obtained by AutoStitch. Two vascular mismatching structures and one blurred structure are indicated by white arrows in (c).

Figure 9 roughly compares the performance of several stitching software by mosaics. In order to evaluate the quality of the mosaic image more accurately, the mosaic image is magnified for comparison, as shown in Figures 10 and 11. Figures 10 and 11 show two examples of mosaics produced by our method. In the figures, the left images refer to the original images captured from one retina at different angles. The proposed method provides visually appealing mosaic images. Mosaics in Figures 10(b) and 11(b) are constructed from nine and six images, respectively. The retinal vessels in the images are accurately stitched. The mosaic images produced by our method and AutoStitch are visually similar. However, mismatched vascular structures by AutoStitch are found as indicated by the white arrows in Figure 10(c). The corresponding vascular structures of the mosaic image are stitched excellently by the proposed method.

Our method can produce mosaic images with less blur, as shown in the zoomed-in regions in Figure 11(b). By contrast, evident blurring around the vessels can be observed in the mosaic image produced by AutoStitch (Figure 11(c)). No evident blur is observed around the vessels in the mosaic image produced by our method. The contents of the mosaic image produced by our method are more consistent with the original retinal images compared with that produced by AutoStitch.

## 4. Discussion and Conclusion

We construct mosaics from a series of images of the human retina. Previous methods were limited to work with images containing clearly visible vascular structures. However, the vascular structure in the retinal image will often become blurred when the fundus bleeds or becomes tumorous. Thus, for all previous methods, detecting robust features in such a retinal image presents a difficulty. This paper addresses the problem of robust feature detection based on obscure vascular structures of retinal images.

Our paper employs a CNN model [12], which effectively obtains clear vascular structures, to segment vascular structures of retinal images. Then, bifurcations in vascular structure are extracted as features, and different transformation estimations are utilized to warp images into a uniform coordinate system for mosaic image blending. Experimentally, we show the mosaic examples and list a summary of numerical results, such as RMSE, recall rate. The proposed feature detection method is superior to other methods, such as ORB and SURF. The quality of mosaic images produced by our method is superior to that of AutoStitch, photomerge function in Photoshop cs6 and ICE, which demonstrate uneven vascular mismatching. The quality of mosaic images produced by our method is especially valuable for retina change detection. Although visually appealing results can be produced with the proposed method, its clinical usefulness should be further confirmed. We plan to improve the efficiency of the algorithm to realize real-time stitching algorithm. In addition, we plan to improve the algorithm for precise registration on large images with different overlaps.

## Figures and Tables

**Table 1:** Comparison of the running time and the average number of features for 62 images using different feature detection algorithms.

Algorithm	ORB	SIFT	Ours	SURF
Running time (s)	0.152	0.741	0.141	0.255
Average number of features	65	98	256	585

**Table 2:** Performance of feature matching with pairs of images within the data set.

Test sample	Overlap (%)	RMSE (pixel)
T01-02	24.9	2.85
T03-04	33.1	1.61
T05-06	36.6	2.63
T07-08	38.4	1.09
T09-10	39.1	1.59
T11-12	52.4	1.35
T13-14	57.3	1.20
T15-16	61.2	2.07
T17-18	61.3	1.66
T19-20	67.3	1.40
Mean value	47.16	1.745

**Table 3:** Average computation time and the average number of matching points for different algorithms.

Algorithm	ORB	SIFT	Ours	SURF
Running times (s)	0.149	0.259	0.249	0.689
Average number of matching points	29	17	62	42

## Data Availability

The data used to support the findings of this study are included within the article and also available from the corresponding author upon request.
